# The Potential of Heavy-Ion Therapy to Improve Outcomes for Locally Advanced Non-Small Cell Lung Cancer

**DOI:** 10.3389/fonc.2017.00201

**Published:** 2017-09-05

**Authors:** Stephen G. Chun, Timothy D. Solberg, David R. Grosshans, Quynh-Nhu Nguyen, Charles B. Simone, Radhe Mohan, Zhongxing Liao, Stephen M. Hahn, Joseph M. Herman, Steven J. Frank

**Affiliations:** ^1^Department of Radiation Oncology, Division of Radiation Oncology, The University of Texas MD Anderson Cancer Center, Houston, TX, United States; ^2^Department of Radiation Oncology, University of California San Francisco, San Francisco, CA, United States; ^3^Maryland Proton Therapy Center, University of Maryland Baltimore, Baltimore, MD, United States

**Keywords:** carbon, heavy ion, locally-advanced lung cancer, hypofractionation, immune therapy

The treatment of unresectable locally advanced non-small cell lung cancer (LA-NSCLC) remains a daunting challenge. To date, the best median survival achieved in a randomized prospective bi-modality clinical trial for LA-NSCLC is 28.7 months for patients who received standard chemoradiotherapy on NRG Oncology RTOG 0617 (1). Emerging data from RTOG 0617 and other institutions have also implicated radiation dose to the heart as a driver of cardiovascular events, survival, and patient-reported quality of life (2–5). To improve survival and quality of life for LA-NSCLC, it is critical to explore emerging therapeutic technologies. One such technology is radiation therapy delivered with heavy ions, such as carbon ions. Heavy-ion therapy has both unique biological and physical advantages that may improve local control while also reducing radiation exposure to non-target organs at risk such as the heart. With the technology implemented at several Asian and European centers in conjunction with the planned development of several therapeutic heavy-ion centers in the United States, there will be real opportunity to exploit this technology to gain ground and improve the therapeutic ratio in LA-NSCLC.

LA-NSCLC is highly resistant to conventionally fractionated radiation therapy with cooperative group studies showing locoregional failure rates greater than 60% after definitive chemoradiation (6). From a radiation biology standpoint, there is considerable rationale to support use of heavy-ion therapy to improve local control for these patients. Upon encountering target tissue, interaction of charged heavy ions with matter yields the highest linear energy transfer (LET) of any currently available form of clinical radiation (7). This in turn results in unique clustered DNA lesions, resulting in a lower oxygen enhancement ratio and higher relative biological effectiveness (RBE) that is on the order of threefold greater than photon radiotherapy (7). Early preclinical data have also suggested tumors with EGFR mutation may be more susceptible to heavy-ion therapy than photon irradiation, suggesting that heavy-ion therapy may provide opportunities to further tailor radiation therapy in the heterogeneous genetic landscape of LA-NSCLC (8). Taken together, these properties suggest that heavy ions could improve local control by overcoming DNA repair pathways that confer radiation resistance and provide patient-tailored options for LA-NSCLC.

The physical properties of heavy-ion therapy also provide dosimetric advantages for LA-NSCLC (9). With particle therapy, the energy deposited in tissue increases with depth eventually coming to an abrupt stop, known as the Bragg peak. Using particles, practitioners have the ability to stop the dose deposition at a specified point, reducing or eliminating exposure to tissues distal to the target volumes. These advantages are particularly relevant to recent findings in RTOG 0617 where the importance of radiation conformity to prevent pneumonitis and reduce cardiac doses has been established (10, 11). Just as proton therapy can reduce cardiac doses in comparison to photon therapy (12), heavy ions also have the potential to further reduce cardiac and pulmonary doses (9, 13). First, the Bragg Peak can reduce radiation doses distal to the target to a far greater degree than photon irradiation. Second, heavy ions exhibit less scattering than protons because of their mass, resulting in a sharper lateral penumbra than proton or photon radiotherapy. Third, pencil beam scanning and arc technologies are expected to provide unprecedented geometric avoidance of non-target organs at risk. The combination of these physical advantages with motion management and Monte Carlo algorithms for plan optimization has potential to produce dramatic improvements in the conformity of the high, intermediate, and low dose regions. While proton therapy exhibits some of these characteristics such as the Bragg Peak, proton therapy has more lateral scattering and lacks the LET of heavy-ion therapy.

In the era of anti-PD-1-/PDL-1-targeted therapies, another important biological advantage of heavy-ion therapy is its potential immunostimulatory effects. While radiation therapy is known to have complex reactions with the immune system and tumor microenvironment, heavy ions have may have unique immune effects that are distinct from photons or proton therapy. Because of their high LET and RBE, preclinical evidence suggests that heavy ions induce non-apoptotic cell death that is independent of the typical p53, bax/bcl, and p21 signal transduction cascades (14, 15). These alternative forms of cell death could provide more diverse tumor epitopes for cytotoxic T-cells to prime immune responses. Another immunological advantage of heavy-ion therapy stems from the unique physical properties discussed earlier. Reducing low and intermediate dose exposure has the potential to reduce integral dose that can cause lymphopenia and hematologic toxicity (16–18). In RTOG 0617, Grade 3+ hematological toxicity was observed in 56% of patients with only one-third of patients completing consolidative chemotherapy (1), highlighting the need to reduce the myelosuppressive effects of definitive chemoradiotherapy. Thus, by reducing lymphopenia from exposure of circulating lymphocytes and also stimulating the local production of immunological epitopes, heavy-ion therapy might be a potent weapon to illicit *in situ* vaccine responses.

Conventionally fractionated radiation therapy as a single modality has dismal cure rates (19), and the primary benefit of concurrent chemotherapy in historic combined modality trials has been to improve local control (20–22), which comes at the cost of myelosuppression, esophagitis, and peripheral neuropathy in LA-NSCLC. However, heavy-ion therapy can deliver doses to tumor targets with greater potency than concurrent chemoradiation without the systemic side effects of chemotherapy. This raises the question of whether heavy-ion therapy can obviate the need for concurrent cytotoxic therapy. Concurrent chemotherapy has significant downsides including high rates of severe esophagitis and immunosuppression. A Phase I–II of carbon ion therapy for patients with LA-NSCLC who were medically unfit to receive concurrent chemotherapy from the Research Institute for Charged Particle Therapy in Chiba, Japan showed promising results (23). In this study, dose was escalated from 68 to 72 cobalt Gy equivalents without dose limiting toxicity. Oncological outcomes were also favorable with a 2-year local control rate of 93.1% and overall survival of 51.9%. There has also been success using carbon ion therapy without cytotoxic chemotherapy for medically inoperable early-stage NSCLC (13, 24–26). Exploration of heavy-ion technology as a strategy to avoid concurrent cytotoxic chemotherapy should be encouraged as a way to reduce toxicities and health-care costs for patients without compromising oncological efficacy.

Currently used fractionation schedules for carbon therapy differ greatly from standard photon techniques. Given differential biological effects on tumors normal tissues as a function of dose per fraction, hypofractionated approaches are commonplace in the practice of heavy-ion therapy. The use of hypofractionated treatment schedules may allow for a greater number of patients to be treated at select centers at a reduced cost. Coupled with the potential for reduced toxicity and improved outcomes, this could make heavy-ion therapy a cost effective treatment, despite the high upfront costs of building such facilities likely on the order of $200–300 million (USD).

As the number of heavy-ion therapy centers is expected to increase in the United States in the coming years, there will be opportunity to explore its role for LA-NSCLC. We have outlined rationale for robust exploration of heavy-ion therapy in LA-NSCLC for the purpose of improving what are presently suboptimal outcomes in this population.

## Author Contributions

All authors have contributed to conceptualization and writing of this manuscript.

## Conflict of Interest Statement

The authors declare that the research was conducted in the absence of any commercial or financial relationships that could be construed as a potential conflict of interest.
